# SARS-Cov-2 trajectory predictions and scenario simulations from a global perspective: a modelling study

**DOI:** 10.1038/s41598-020-75332-x

**Published:** 2020-10-27

**Authors:** Tianan Yang, Yexin Liu, Wenhao Deng, Weigang Zhao, Jianwei Deng

**Affiliations:** 1 School of Management and Economics, Beijing Institute of Technology, Beijing, China; 2 Sustainable Development Research Institute for Economy and Society of Beijing, Beijing, China; 3 Center for Energy and Environmental Policy Research, Beijing Institute of Technology, Beijing, China

**Keywords:** Diseases, Medical research

## Abstract

The coronavirus SARS-CoV-2 emerging from Wuhan, China has developed into a global epidemic. Here, we combine both human mobility and non-pharmaceutical interventions (social-distancing and suspected-cases isolation) into SEIR transmission model to understand how coronavirus transmits in a global environment. Dynamic trends of region-specific time-variant reproduction number, social-distancing rate, work-resumption rate, and suspected-cases isolation rate have been estimated and plotted for each region by fitting stochastic transmission processes to the real total confirmed cases reported of each region. We find after shutdown in Wuhan, the reproduction number in Wuhan greatly declined from 6·982 (95% CI 2·558–14·668) on January 23rd, 2020 to 1.130 (95% CI 0.289–3.279) on February 7th, 2020, and there was a higher intervention level in terms of social-distancing and suspected-cases isolation in Wuhan than the Chinese average and Western average, for the period from the shutdown in Wuhan to mid-March. Future epidemic trajectories of Western countries up to October 10th, 2020, have been predicted with 95% confidence intervals. Through the scenario simulation, we discover the benefits of earlier international travel ban and rigorous intervention strategies, and the significance of non-pharmaceutical interventions. From a global perspective, it is vital for each country to control the risks of imported cases, and execute rigorous non-pharmaceutical interventions before successful vaccination development.

## Introduction

As a severe acute respiratory syndrome coronavirus, COVID-19 has spread to various countries since the Wuhan outbreak due to its high infectiousness and the lack of effective drugs, which has led to disastrous social and economic difficulties. According to the World Health Organization, more than five million confirmed cases have been recorded worldwide as of May 29th, 2020, indicating that the prevention and control of COVID-19 has reached an urgent point. Considering the globalizing trend of the epidemic, it is imperative to take into account the influence of human mobility on epidemic growth. The traditional SEIR (susceptible-exposed-infected-removed) model that applies in isolated regions may become no longer appropriate to simulate the epidemic changes in a global environment as it ignores the imported cases which pose an increasing risk for each country^1^.

Most previous studies either have simulated scenarios in which the epidemic grows under different governmental interventions without considering human mobility, or have considered human mobility but without taking governmental responses into account^2,3^. We searched PubMed for articles published in English as of May 29th, 2020, with the keywords “COVID-19” in title, keywords “isolation” and “SEIR” in the title or abstract, and totally seven studies were retrieved, but none of them consider regional human mobility; then, we searched with the keywords “COVID-19” in title, keywords “isolation” and “modelling” in the title or abstract, four studies were retrieved, only one of them combines the isolation, social-distancing, and regional human mobility in China^4^.

Thus, this study fills the gap by combining the traditional SEIR model with both non-pharmaceutical governmental interventions, including social-distancing and isolation of suspected cases, with the human mobility between Wuhan, Hubei Province excluding Wuhan, other provinces of mainland China, and thirteen Western countries (Switzerland, Sweden, Austria, France, United Kingdom, Germany, Italy, Spain, Norway, Netherlands, Belgium, Denmark). Consequently, this may lead to a less biased prediction or simulation from a more comprehensive global perspective.

There are four objectives in this study: first, we aim to evaluate the effects of Wuhan shutdown, more specifically: how much of R0 (basic reproduction number) has been reduced by Wuhan shutdown, based on this globalized SEIR model; second, we aim to compare the intervention differences between Chinese and Western regions in terms of social-distancing and suspected-cases isolation in the early period after Wuhan shutdown; third, we aim to reproduce the regional epidemic trajectories from December 10th, 2019 up to May 1st, 2020 and predict trajectories from May 2nd, 2020 to October 4th, 2020; finally, we aim to evaluate the necessity and significance of non-pharmaceutical interventions by simulating different scenarios, which include how the epidemic trajectories of Western countries would change if interventions were executed in the same way as Wuhan from different time-points, how the epidemic would develop if Western governments banned all international human mobility thoroughly from different time-points, and how the epidemic trajectories of Western countries or China would grow if there were none or less non-pharmaceutical interventions in effect.

Overall, this study complements modelling studies on the dynamic trajectory of COVID-19 and provides a tool for policy-simulation which can assist countries around the world in evaluating previous interventions and deciding the right time, region, and degree of executing future interventions, ranging from social-distancing, suspected-cases isolation, to mobility bans.

## Results

### Trajectories of region-specific time-variant reproduction numbers

**Figure 1:**
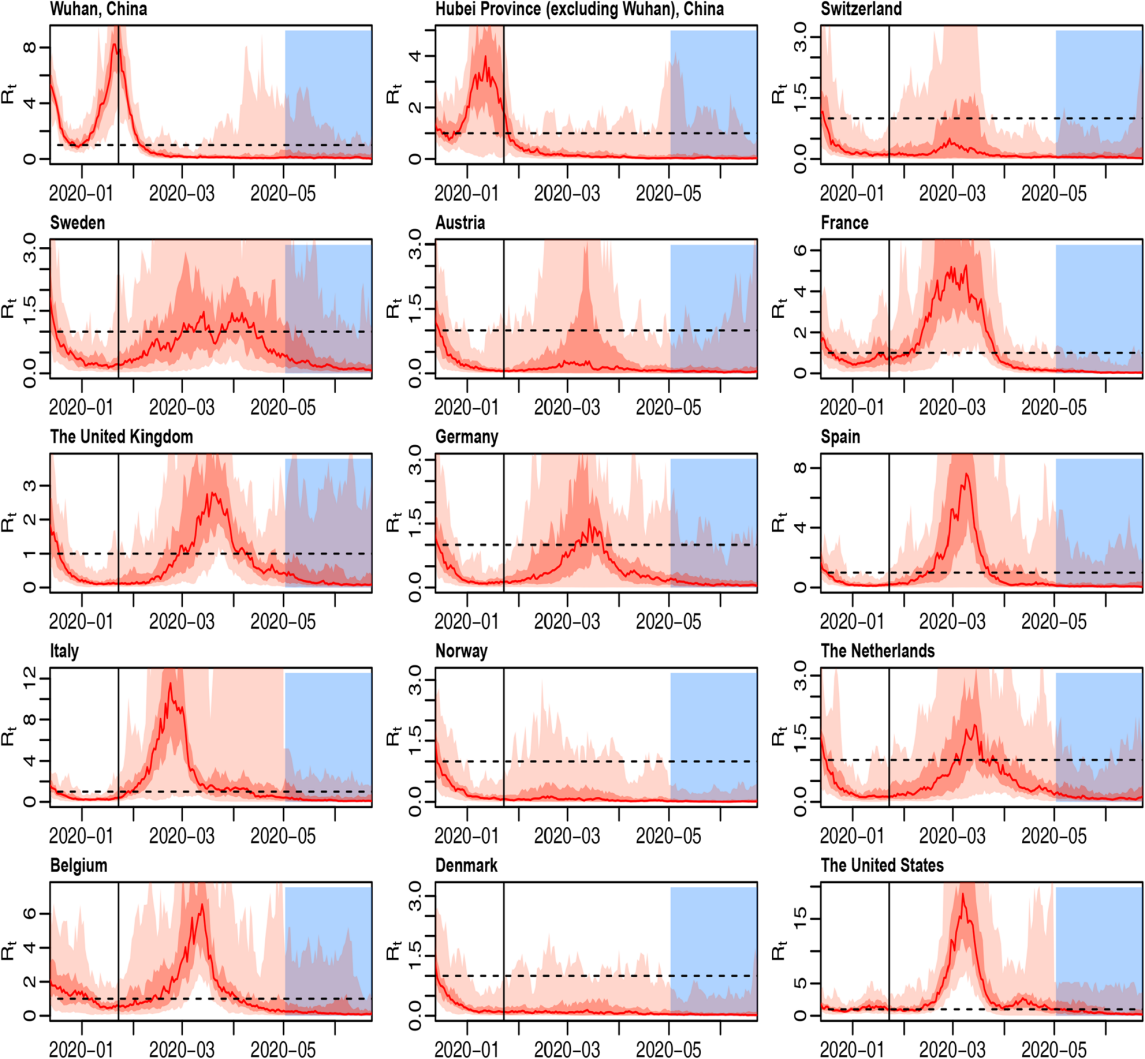
Region-specific time-variant reproduction numbers for fifteen regions, not controlling for under-reporting. The horizontal black imaginary line represents R0 at 1, the vertical black line represents January 23rd, 2020, the date of the shutdown in Wuhan, and the blue area on the right side of the picture represents the predicted period from May 1st to June, 26th, 2020.

**Figure 2:**
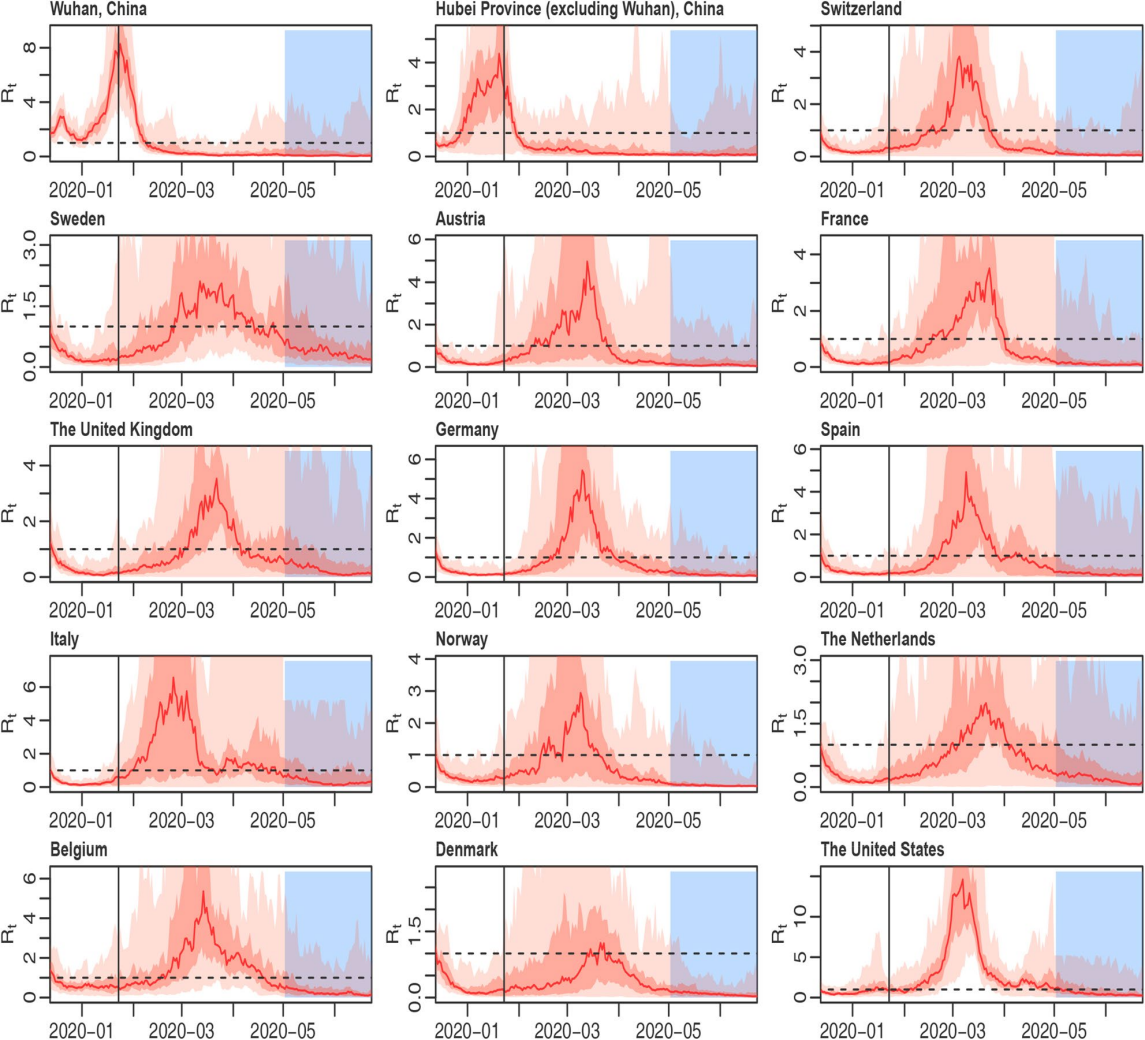
Region-specific time-variant reproduction numbers for fifteen regions, controlling for under-reporting. The horizontal black imaginary line represents R0 at 1, the vertical black line represents January 23rd, 2020, the date of the shutdown in Wuhan, and the blue area on the right side of the picture represents the predicted period from May 1st to June, 26th, 2020.

Figure 1 displays the trajectories of region-specific time-variant reproduction numbers (R0) for fifteen regions (Wuhan, Hubei Province excluding Wuhan, Switzerland, Sweden, Austria, France, the United Kingdom, Germany, Spain, Italy, Norway, the Netherlands, Belgium, Denmark, and the United States), not controlling for under-reporting. For each subplot, the red trajectory represents the 50% quantile of reproduction number distribution, while two shades of light red represent the interval between 25 and 75% quantiles and 2.5–97.5% quantiles of reproduction number distribution, respectively. The blue region represents predicted trajectory after May 1st, 2020. After shutdown in Wuhan, the reproduction number in Wuhan greatly declined from 6.982 (95% CI 2.558–14.668) on January 23rd, 2020 to 1.130 (95% CI 0.289–3.279) on February 7th, 2020. And for almost all Western countries, the reproduction numbers peaked in mid-March, and then declined gradually. For example, in the United States, reproduction number declined from 12.510 (95% CI 5.606–39.015) on March 10th, 2020 to 1.909 (95% CI 0.336–4.303) on March 30th, 2020, then to 1.113 (95% CI 0.051–5.216) on May 1st, 2020. Region-specific time-variant reproduction numbers for fifteen regions controlling for under-reporting have been drawn in Fig. 2. After controlling for under-reporting, the effect of Wuhan shutdown remains consistent, and the R0 trajectories become much steeper with a higher peak for the United States, France, Spain, and Italy from March to May, and become much flatter with a lower peak for Norway, Sweden, Germany, Switzerland, and Denmark, which implies that after controlling for under-reporting, more cases in the lower-peak countries may be imported from the higher-peak countries.

### Trend plots of region-specific time-variant social-distancing rate, suspected-cases isolation rate, work-resumption rate and accumulated imported cases from abroad

**Figure 3:**
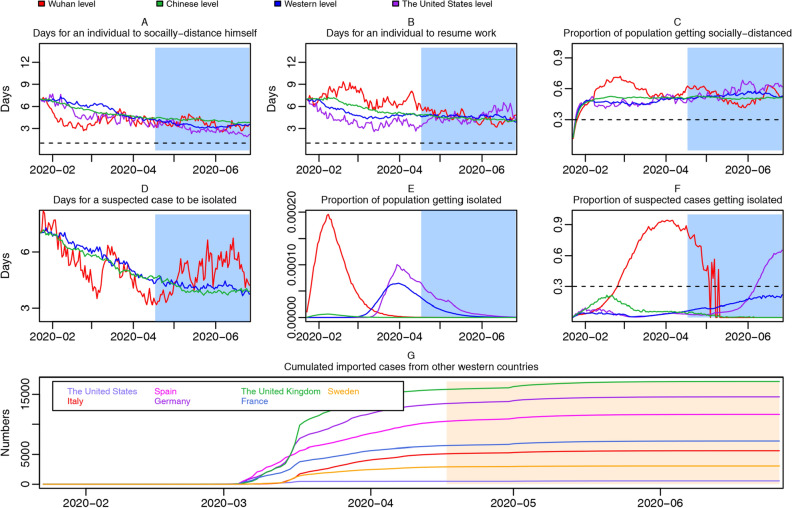
Trend plots of region-specific time-variant social-distancing rate, suspected-cases isolation rate, work-resumption rate and accumulated imported cases from abroad, not controlling for under-reporting. Social-distancing rate trajectories for Wuhan, the Chinese average, the United States, and the Western average, with the horizontal dotted line representing 1 day (**A**); work resumption rate trajectories for Wuhan, the Chinese average, the United States, and the Western average, with the horizontal dotted line representing 1 day (**B**); proportion of socially-distanced people in susceptible population for Wuhan, the Chinese average, the United States, and the Western average, with the horizontal dotted line representing 30% (**C**); isolation rate trajectories for Wuhan, the Chinese average, and the Western average (**D**); proportion of population getting isolated in Wuhan, the Chinese average, the United States, and the Western average (**E**); proportion of suspected cases getting isolated in Wuhan, the Chinese average, the United States, and the Western average, with the horizontal dotted line representing 30% (**F**); accumulated imported cases from other Western countries (**G**). Under-reporting is not controlled for.

Trend plots of region-specific time-variant social-distancing rate, suspected-cases isolation rate, work-resumption rate, and accumulated imported cases are drawn in Fig. 3 (not controlling for under-reporting).

In Fig. 3A, during most time from January 23rd, 2020 to April 1st, 2020, days needed for an individual to become fully socially-distanced in Wuhan, are less than Chinese average, followed by Western average, and then by the United States, suggesting a higher social-distancing rate in Wuhan, manifesting citizens were acting more rapidly to increase social-distancing and reduce face-to-face contacts.

In Fig. 3B, days needed for a socially-distanced individual to resume work in Wuhan are more than Chinese average, followed by Western average, and then by the United States, suggesting a lower work-resumption rate in Wuhan. However, the social-distancing rate in the United States is gradually increasing, suggesting the United States government was making every effort to control the social distancing of citizens. It is not surprising to see that work-resumption rate in Wuhan increases gradually since early March, suggesting a regressing epidemic and a progressing work resumption in Wuhan to reinvigorate the economy^5^.

The result in Fig. 3C, or the higher peak of red curve (Wuhan), further supports previous discoveries: possibly due to rigorous inter-city and inter-community shutdown, more susceptible people in Wuhan had been social-distanced since early February.

As shown in Fig. 3D, for most of time, the isolation rate of suspected cases in Wuhan was consistently higher than Chinese and Western average (the number of days needed for a suspected case to be isolated in Wuhan were less), indicating a more efficient epidemiological contract tracing. However, all regions were experiencing an increasing speed in isolation, pointing out that both Chinese and Western governments were donating jointed efforts to isolate suspected cases.

Possibly due to a more rapid and rigorous intervention, for one side, proportion of isolated cases in whole population peaked earlier in February and higher for Wuhan than for the Western average and the United States, as that of latter peaked after April (Fig. 3E), and for the other side, proportion of isolated cases in suspected cases also grew more rapidly for Wuhan (Fig. 3F), where about 95% suspected cases had been isolated up to April 1st. And proportion of suspected cases getting isolated in the United States increased sharply after June.

Figure 3G displays how many cases were imported from abroad for Western countries from January 23rd, 2020, to July 1st, 2020. The first three Western countries with the most accumulated imported cases from abroad are respectively the United Kingdom, Germany, and Spain, with 3258 (95% CI 652–188,472), 2859 (95% CI 552–181,195), and 2739 (95% CI 519–166,091) imported cases by May 1st, 2020, respectively.

**Figure 4:**
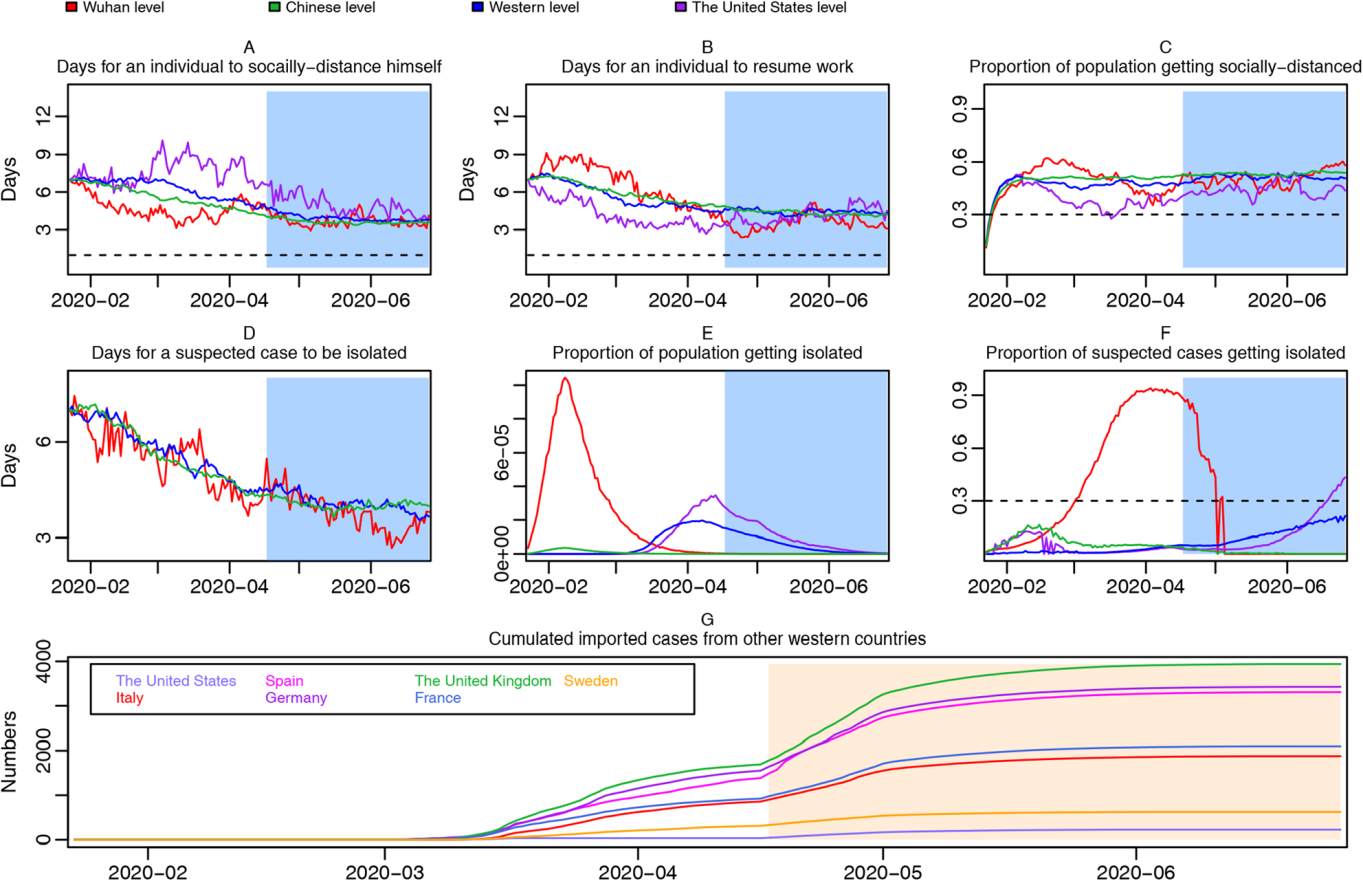
Trend plots of region-specific time-variant social-distancing rate, suspected-cases isolation rate, work-resumption rate and accumulated imported cases from abroad, controlling for under-reporting. Social-distancing rate trajectories for Wuhan, the Chinese average, the United States, and the Western average, with the horizontal dotted line representing 1 day (**A**); work resumption rate trajectories for Wuhan, the Chinese average, the United States, and the Western average, with the horizontal dotted line representing 1 day (**B**); proportion of socially-distanced people in susceptible population for Wuhan, the Chinese average, the United States, and the Western average, with the horizontal dotted line representing 30% (**C**); isolation rate trajectories for Wuhan, the Chinese average, and the Western average (**D**); proportion of population getting isolated in Wuhan, the Chinese average, the United States, and the Western average (**E**); proportion of suspected cases getting isolated in Wuhan, the Chinese average, the United States, and the Western average, with the horizontal dotted line representing 30% (**F**); accumulated imported cases from other Western countries (**G**). Under-reporting is controlled for.

Trend plots yet controlling for under-reporting are drawn in Fig. 4. Most discoveries remain consistent except the social-distancing rate of the United States becomes higher, and the peak of population getting isolated arrived earlier for the United States. In Fig. 4G, we can see that accumulated imported cases for Western countries increased sharply after controlling for under-reporting: for instance, that in the United Kingdom drastically increased to 16,312 (95% CI 1704–180,594) by May 1st.

### Trajectory predictions

**Figure 5:**
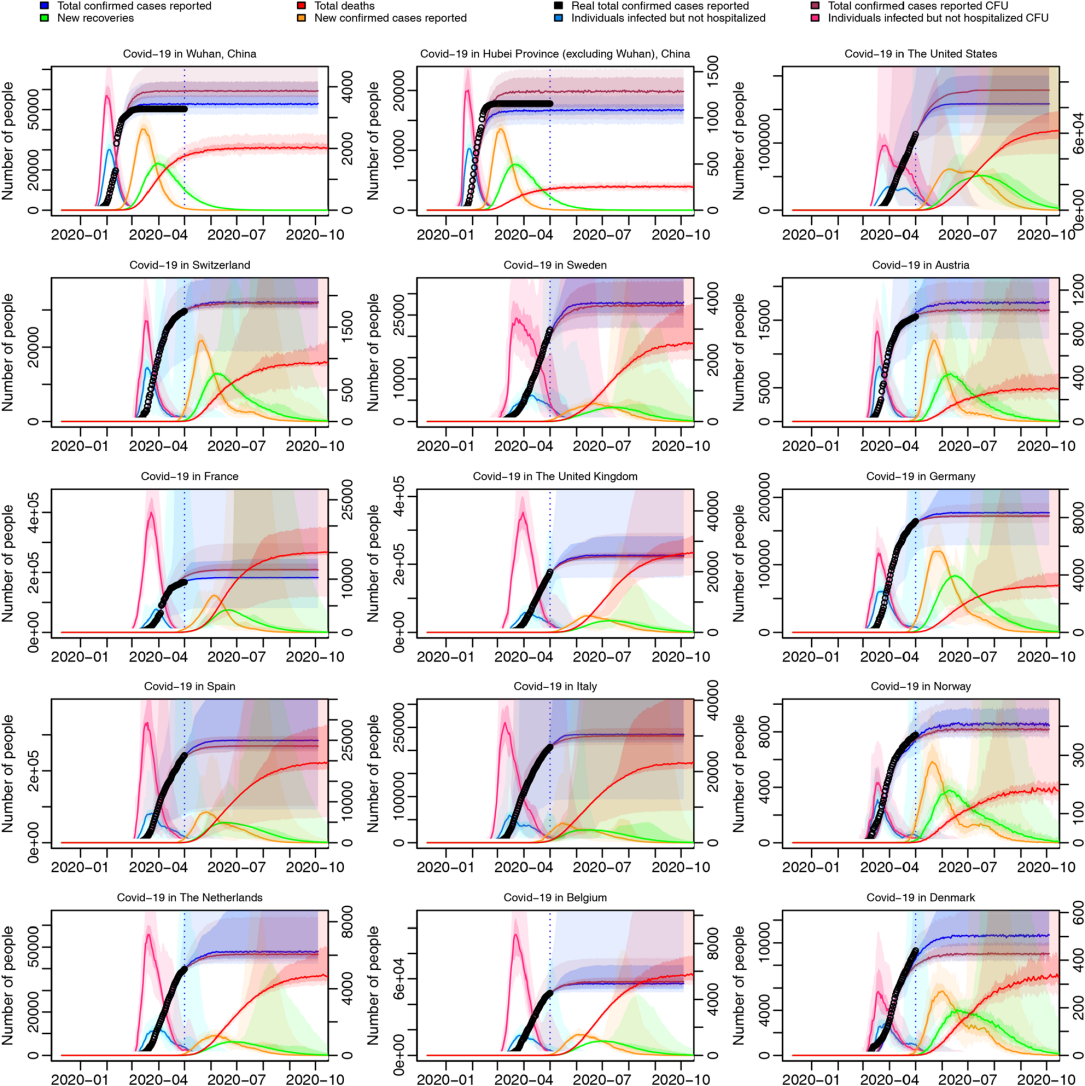
Epidemic trajectories of Wuhan, Hubei Province excluding Wuhan, and thirteen Western countries. Total number of confirmed cases reported, controlling for under-reporting (purple curve, left axis) and not controlling for under-reporting (blue curve, left axis); total number of individuals infected but not hospitalized, controlling for under-reporting (pink curve, left axis) and not controlling for under-reporting (sky-blue curve, left axis); new confirmed cases (orange curve, right axis), total deaths (red curve, right axis), and new recoveries (green curve, right axis) every day not controlling for under-reporting; All trajectories are predicted from May 2nd, 2020 to October 4th, 2020, for Wuhan, Hubei Province (excluding Wuhan), and thirteen Western countries, based on total-confirmation real data, all with 50% and 95% CI. Black point refers to historical real total number of confirmed cases reported per day. CFU means “controlling for under-reporting”.

The trajectory predictions are displayed in Fig. 5. For all regions, after controlling for under-reporting, newly increased individuals infected but not hospitalized (pink curve, left axis) grows to a higher level, compared with not controlling for under-reporting (sky-blue curve, left axis), which reflects that there are possibly much more infected individuals not hospitalized than originally expected in the early period of epidemic. However, total cases reported controlling for under-reporting (purple curve, left axis) do not differ saliently from not controlling for under-reporting for most regions, which means many unreported cases either recover or die before reporting. The trajectories for the total number of confirmed cases in Wuhan and Hubei Province (excluding Wuhan) stopped growing at the beginning of March even after controlling for under-reporting (purple curve, left axis), indicating that the epidemic growth was gradually getting controlled in China at that time. For most western countries, from the trajectories of newly increased confirmed cases reported (orange curve, right axis), we can see that the epidemic inflection-point would approach around the middle of May or the early June, 2020, while the peak of trajectories of new recoveries (green curve, right axis) would come around the middle of June, 2020, but the inflection points of the trajectories of total deaths (red curve, right axis), seem to come after August, which is reasonable considering that there is a delay between confirmations and recoveries or deaths.

### Scenario simulations

**Figure 6:**
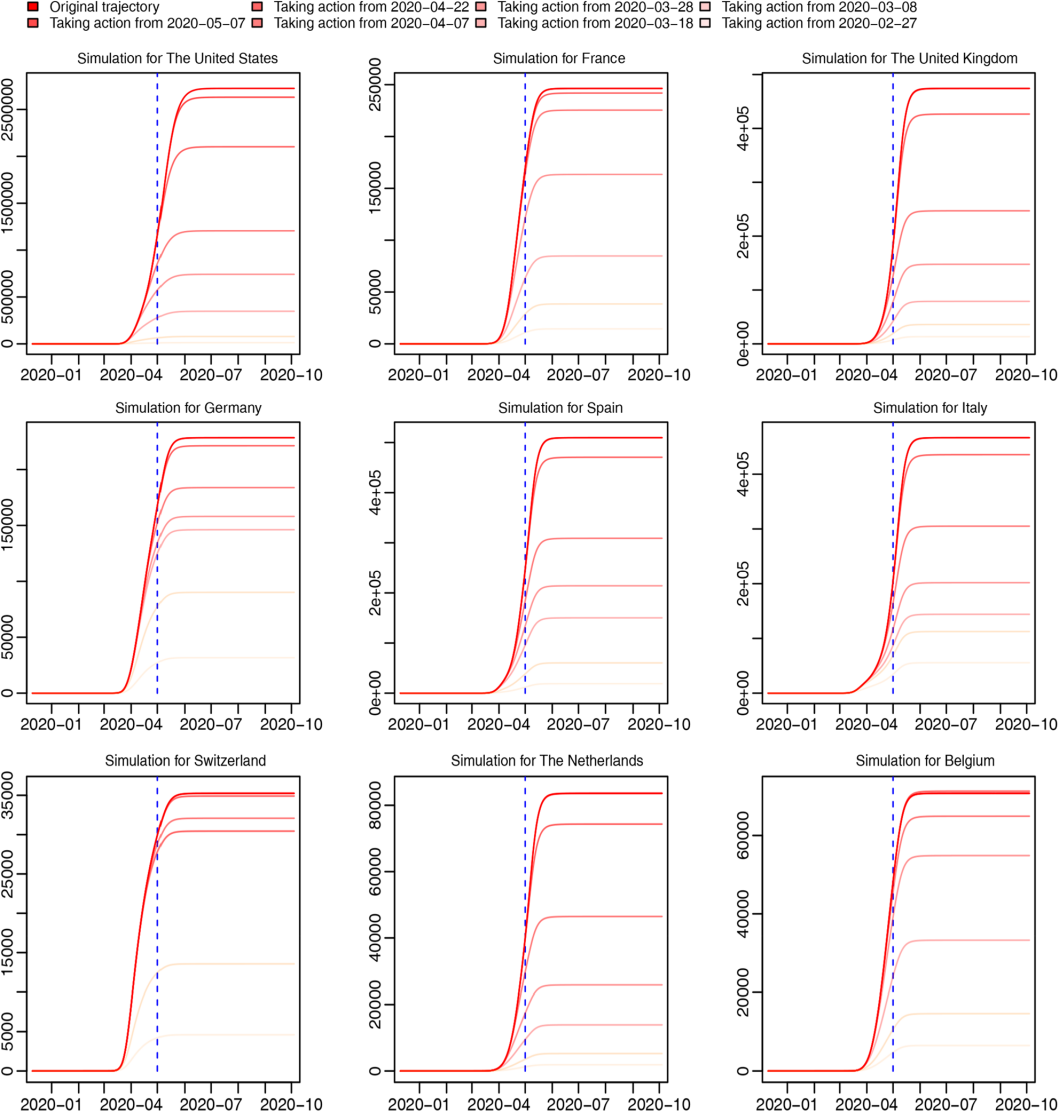
Simulations of the first scenario. Curves of different colors represent trajectories of total confirmed cases reported for nine randomly selected Western countries in different occasions where NPI of western countries were executed in the same way as Wuhan from different time points. The deeper the color is, the later Wuhan-level interventions were executed. Different time-points to take action are exhibited in the legend.

For the first scenario (Fig. 6), We discover that the earlier Western governments carry out NPI (non-pharmaceutical interventions), namely, social-distancing and suspected-cases isolation, in the same way as Wuhan (the average Wuhan level of parameters during the first month after the shutdown in Wuhan), the smaller the total number of confirmed cases there will be, while the later, the less benefits.

**Figure 7:**
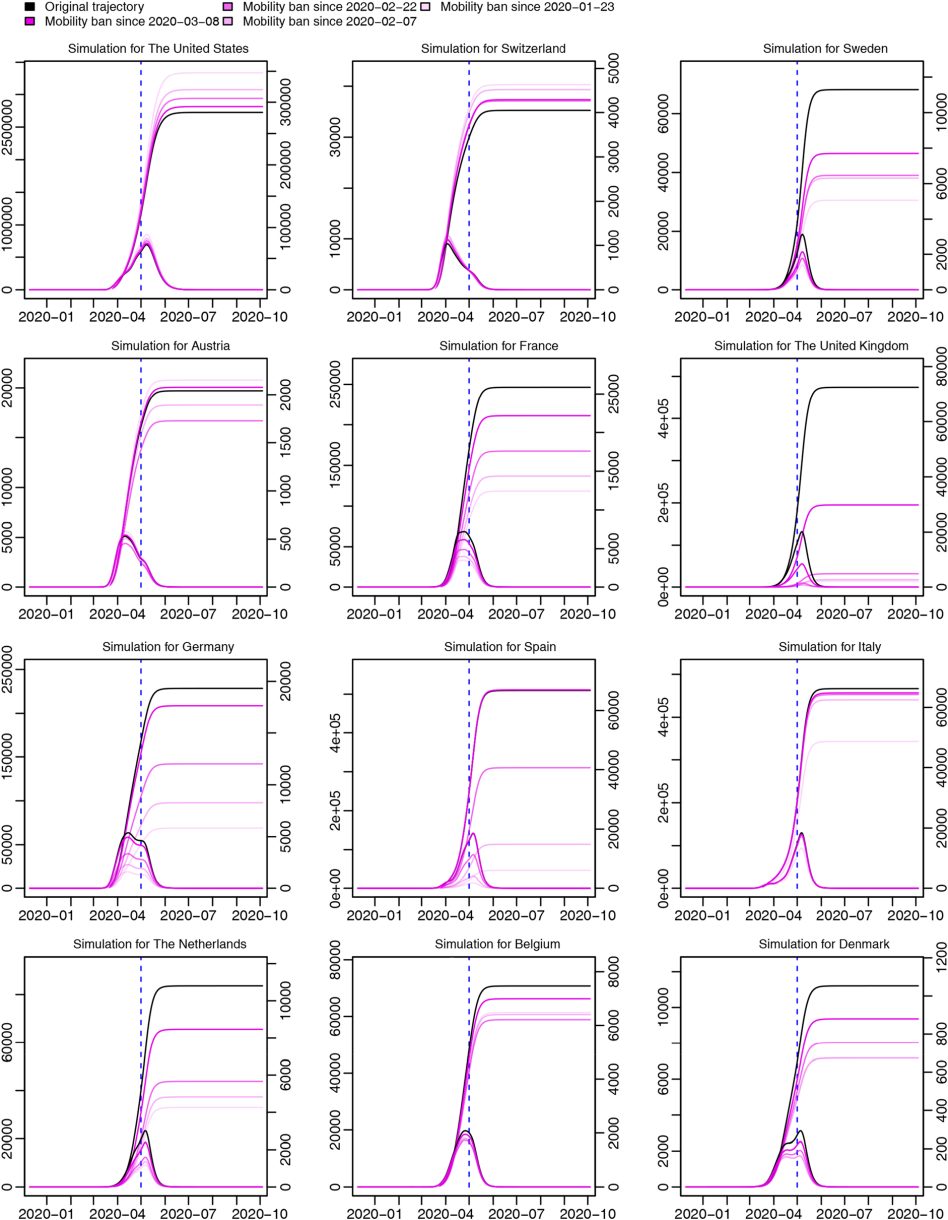
Simulations of the second scenario. Curves of different colors represent trajectories of total confirmed cases reported every day (left axis) and new confirmed cases reported every day (right axis) for twelve randomly selected Western countries in different occasions where complete international travel ban were executed from different time points. The deeper the color is, the later travel bans were executed. Different time-points to take complete international travel ban are exhibited in the legend.

For the second scenario (Fig. 7), we first discover that for most Western countries, it is always better to take complete international travel ban as early as possible, but for the United States and Switzerland, earlier travel ban seemingly works in the opposite way: possibly in the early period of epidemic, at least in our model, there are more infected people moving out from the United States or Switzerland to other countries, than from other countries moving back, through which mobility bans may lead to more cases; second, though for most Western countries, the earlier mobility is banned, the less the total number of confirmed cases will be recorded in the end, the degree of reduction greatly differs between Western countries: for instance, the effect of earlier mobility ban is more salient in Spain and the United Kingdom, than in Italy, Belgium, and Austria.

**Figure 8:**
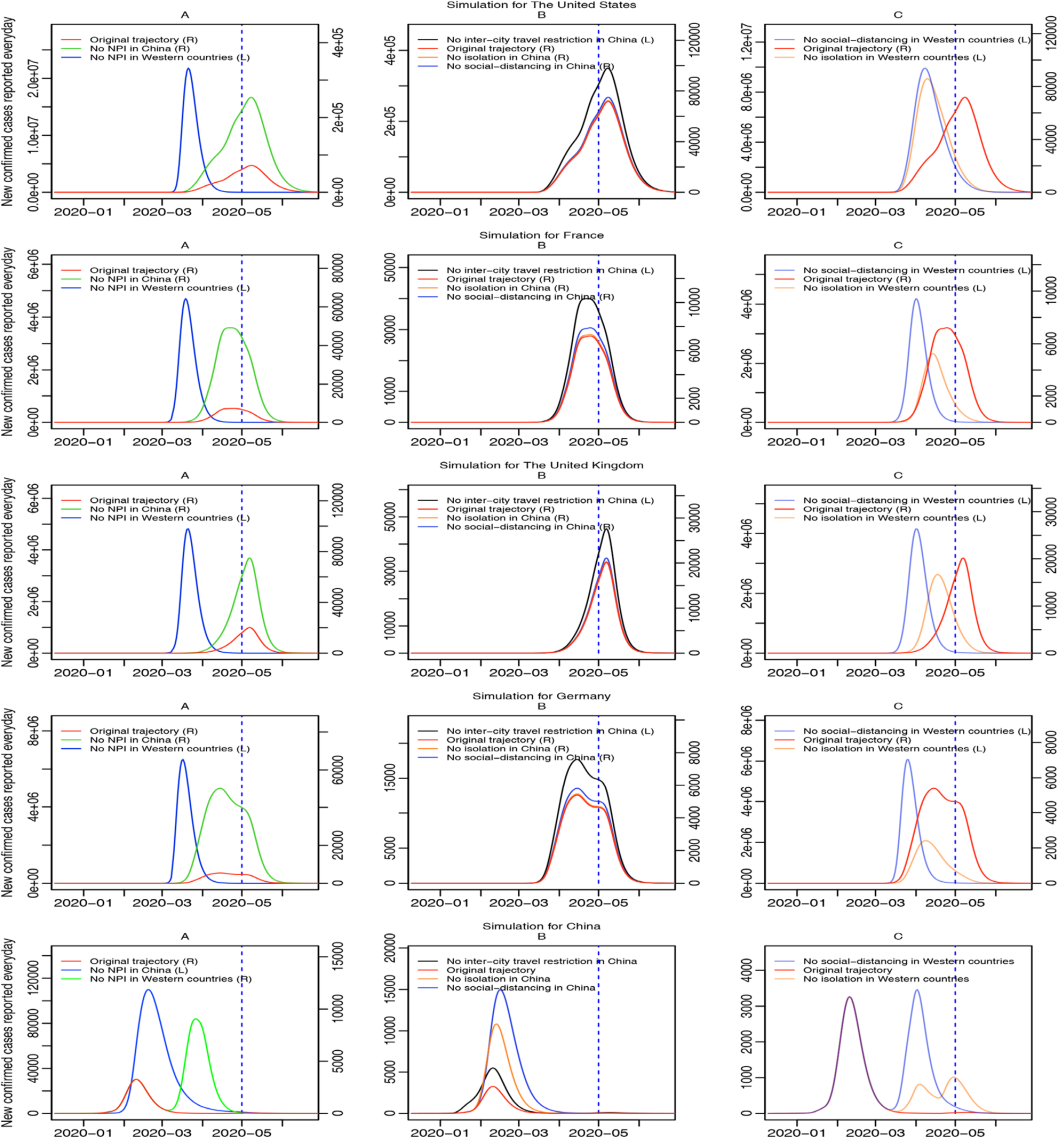
Simulations of the third scenario. Curves of different colors represent trajectories of daily new confirmed cases reported for four selected Western countries and China in different occasions where less or none NPI were executed from January 23rd, 2020. Subplot A draws scenarios that none NPI were executed in either China or Western countries; subplot B draws scenarios that one kind of NPI was not executed in China; and subplot C draws scenarios that one kind of NPI was not executed in Western countries. In the legend, (L) means being drawn on the left axis and (R) means being drawn on the right axis. The blue dotted line represents May 1st, 2020, the final day of fitting.

For the third scenario (Fig. 8), in the subplot A of Western countries, results of green and red curve show if none NPI, including social-distancing, suspected cases isolation, and inter-city travel restrictions was executed in China, compared with the original trajectory (red curve, right axis), the peak of new confirmed cases reported every day (green curve, right axis) would be magnified by about three times for most Western countries, while if there was none NPI in Western countries, the peak of new confirmed cases reported every day (blue curve, left axis) would be magnified by at least one hundred times and the epidemic inflection point would come earlier. More specifically, if all NPI were executed normally in Western countries and there were less NPI in China (shown in subplot B of Western countries), cases of Western countries would be influenced most by no inter-city travel restrictions in China (black curve, left axis), followed by no social-distancing in China (blue curve, right axis) or no isolation in China (orange curve, right axis). Similarly, in subplot C of Western countries, if only one of NPI was not executed in Western countries but all NPI were executed in China, the effect of no social-distancing (blue curve, left axis) would be more serious than no isolation for Western countries (orange curve, left axis), while both of them would cause a significant increase in cases of original trajectories by at least one hundred times.

Finally, for China, in subplot A, if there was none NPI in China and all NPI were executed in Western countries, the peak of new confirmed cases reported every day (blue curve, left axis) would be thirty times higher than that of original trajectory, and if there was none NPI in Western countries and all NPI were executed in China, the peak of new confirmed cases reported every day (green curve, right axis) would be three times higher than that of original trajectory. In subplot B of China, no social-distancing (blue curve), no suspected-cases isolation (orange curve), and no inter-city travel restrictions (black curve) would raise the peak of new confirmed cases reported every day in China by four folds, two folds, and one fold, respectively. In subplot C of China, if all NPI were executed in China, no social-distancing in Western countries would cause a second larger outbreak in April for China (blue curve), and no isolation in Western countries would cause two more outbreaks yet smaller, in April and May (orange curve), respectively.

## Discussion

We develop an extended stochastic meta-population model based on real data from December 10th, 2019 to May 1st, 2020, considering real human mobility in 45 regions (including 32 Chinese regions and 13 Western countries). Differences in the effects of NPI are explored between China and Western countries, including social-distancing and isolation of suspected cases. We find a higher social-distancing rate and a lower work-resumption rate in Wuhan during the time from the shutdown of Wuhan to mid-March, which leads to a higher socially-distanced proportion of Wuhan. Similarly, the suspected-cases isolation rate in Wuhan is higher than the Chinese average and the Western average during most time, leading to a higher proportion of isolated cases in both the whole population and suspected cases (Figs. 3 and 4). We believe the differentiation between Wuhan and the Western average in the effects of NPI results from stricter governmental regulations. In the trajectory prediction (Fig. 5), we discover that after controlling for under-reporting, the individuals infected but not hospitalized in Western countries on a given day would grow drastically, and as most of them would keep infecting others before self-recovery or death, the real infected cases may be far beyond what have been reported. Through the scenario simulations, we conclude that the sooner the Western governments adopt the same level of NPI as Wuhan in China, the sooner the epidemic can be controlled (Fig. 6). In addition, we find that for most Western countries, the earlier international human-mobility bans are implemented, the more cases could be avoided (Fig. 7), suggesting the effectiveness of mobility bans during the early period. However, we do find that for the United States, mobility bans always lead to more local cases, which may be because more cases are moving out of the United States rather than moving in. Finally, we test the effectiveness of non-pharmaceutical interventions, and discover that without such interventions, the epidemic in Western countries could grow up by hundreds of times (Fig. 8), strongly supporting the necessity and significance of non-pharmaceutical interventions, especially before any vaccine inventions. Moreover, without inter-city travel restrictions such as Wuhan shutdown, the epidemic in China and Western countries would increase sharply. Our demonstrations of the effectiveness of social-distancing, suspected-cases isolation, and inter-city travel restrictions are consistent with previous research^3,4,6^. Considering the COVID-19 epidemic is quickly developing into a global crisis, it is urgent to develop a more open model to understand the transmission dynamics of the epidemic from a global perspective^7^. The extended stochastic meta-population model of the present study can help evaluate the effectiveness of controlling measures executed in the past, predict the outbreak proclivity for different regions in the future, and also help determine the most effective and economical time and place to execute interventions by visualizing the regional and time differences of intervention effects. In summary, by combining regional mobility and governmental interventions, this model could guide decision-makers in medical-resources allocation and intervention- strategies design.

During this emergency public health crisis, China has shown a strong responsive capacity and intervention effectiveness, which has been praised by the World Health Organization officials^8^, and the reasons behind are worth discussing. It is widely recognized that since the early outbreak of COVID-19 in January 2020 in Wuhan, the local government has adopted many controlling policies to reduce social-mixing, such as closing the city border and the community exchange on January 23rd, 2020, banning almost all personnel face-to-face exchange, and suspending workplace, school, and public transport^9^. In addition, on the same day of Wuhan shutdown, the Chinese government announced that China had entered a national emergency response state and adopted a series of nation-wide controlling measures^10^. Being able to control epidemics through rigorous policies in a short period of time is undoubtedly an important experience that China could give to the international society to combat the epidemic^4,9^.

There are still some limitations for this study. Firstly, due to data availability, daily human mobility data between Western countries are inferred from the monthly or seasonal data of 2019, which could bring bias to the prediction of future epidemic growth and the simulation of mobility bans. However, the overall trend of prediction would not be affected; Secondly, as the result shows, prediction of future total number of reported confirmed cases may change greatly after controlling for under-reporting, like that of the United States. Thus, if there are any significant under-reporting on real data, then the prediction of future cases could be underestimated, and although we have attempted to control for under-reporting in trajectory prediction, the under-reporting degree we used may still be biased as they were estimated without taking into account age-distribution of each country^11^; Thirdly, we have not considered population demographics and meteorological conditions, which should be integrated in a more realistic model^7^; Finally, as we included only thirteen Western countries in the model after considering countries’ geographical distribution, population, and epidemic development, more countries should be included to reflect a more globalized perspective.

We conclude from this study four main points: first, we have proven that Wuhan shutdown is effective by validating the decline of R0 from 6.982 (95% CI 2.558–14.668) on January 23, 2020 to 1.130 (95% CI 0.289–3.279) on February 7 in Wuhan soon after Wuhan shutdown (Fig. 1), and by finding that more cases will appear worldwide without Wuhan shutdown (Fig. 8); second, we have shown that Wuhan has executed a more effective non-pharmaceutical intervention in the early period after Wuhan shutdown by comparing the time-variant parameters between different regions (Figs. 3 and 4); third, we have found that there will be drastically more infected but not yet hospitalized individuals after controlling for under-reporting for all regions (Fig. 5); and finally, we have proven that non-pharmaceutical interventions are effective and necessary (Figs. 6, 7, 8), especially in the early period of epidemic outbreak, which is right now still meaningful for each nation as there is still the possibility of a sudden local epidemic outbreak, given that COVID-19 will coexist with human for long and vaccines are not readily available.

## Methods

### Model structure

**Figure 9:**
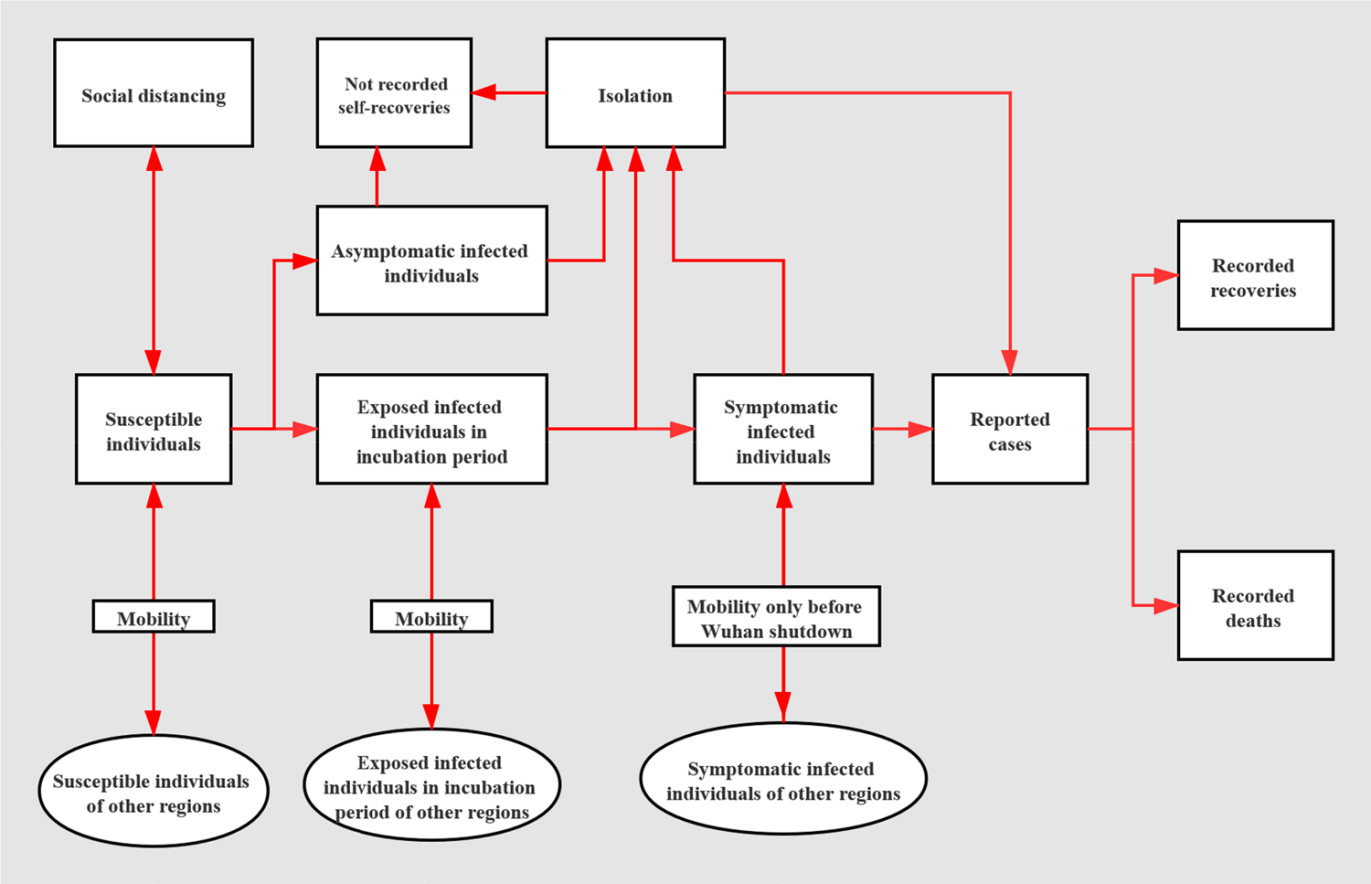
Model structure. The natural infective process is stratified into four stages: susceptible individuals, exposed infected individuals in the incubation period, symptomatic infected individuals, and removed individuals (who either recovered or died). 7.5% asymptomatic cases are assumed. By human mobility, susceptible individuals and exposed infected cases in incubation period could move freely across regions, and symptomatic infected cases could move across regions only before Wuhan shutdown. Susceptible individuals could expand their social distancing to the extent that they would not be infected, at a time-variant social-distancing rate, and similarly, individuals who have social-distanced themselves could resume work at a time-variant work-resumption rate and become susceptible individuals again. Asymptomatic cases, exposed infected individuals in the incubation period, as well as symptomatic infected individuals could be isolated at a time-variant isolation rate. By adjusting human mobility matrix, the international or inter-city travel ban can be realized.

The model structure is displayed in Fig. 9. We stratify the natural infective process into four stages: susceptible individuals, exposed infected individuals in the incubation period, symptomatic infected individuals, and removed individuals (who either recovered or died). Susceptible individuals are those who have not been infected before and are vulnerable to infection, in which they would become exposed infected individuals. After an incubation period of 6.4 days for coronavirus^12^, exposed infected individuals, if not isolated, will develop into symptomatic infected individuals. After a symptom-onset period of 3.8 days^13^, symptomatic infected individuals, if not isolated, would be hospitalized and get tested. A proportion of hospitalized cases would die within an average period of 14.7 days (for non-survivors of coronavirus since hospitalization), and the rest would recover within an average period of 18.2 days (for survivors of coronavirus since hospitalization)^14^. The individuals who recover are assumed to gain immunity and would not be infected again. In our model we assume that healthcare facilities like negative pressure isolation wards and personal protective equipment (PPE) for medical staff are abundant, so that no further infection would occur once infected individuals are hospitalized or isolated. To take asymptomatic cases into account, we assume 7.5% of cases would be asymptomatic before recovery^15^, who will remain at the stage of exposed infected individuals and keep infecting others with the same infectiousness of symptomatic cases until being isolated or self-recovered^16^. Before being reported, exposed infected individuals or symptomatic infected individuals are suspected cases.

After individuals are hospitalized, official reporting will be performed. Considering the average testing and official reporting speeds for Wuhan were different before and after January 27th, 2020, we assume days required for reporting individual cases in China are 4.5 days and 2.8 days before and after January 27th, 2020^13^. For western countries, considering the difficulty in confirming unknown COVID-19 cases before the Wuhan outbreak, we assume the reporting rate would gradually lineally accelerate to 4.5 days by February 19, and then lineally accelerate to 2.8 days by March 8th, to reflect a growth of public awareness and government response. Delays are accounted for between infection, symptom onset, hospitalization, and recovery, by dividing the corresponding processes into two compartments^17^. We assume cases would have no infectiousness until the second phase of exposed infection so that the infectivity of symptomatic cases would be about twice that of exposed cases in the incubation period^18^.

We integrate human mobility process into traditional SEIR model to consider daily human mobility, through which susceptible individuals, exposed infected individuals in the incubation period, and symptomatic infected individuals could be imported and exported freely between regions from December 10th, 2019 to January 23rd, 2020, the time of Wuhan shutdown. However, after the shutdown in Wuhan, we assume only susceptible individuals and exposed infected individuals could travel freely by assuming symptomatic infected individuals would be precisely screened out.

For governmental interventions, besides the estimation and drawing of region-specific time-variant reproduction numbers, we also estimate region-specific time-variant social-distancing rate, work-resumption rate, and suspected-cases isolation rate by fitting stochastic process containing 2000 operators to the real total confirmation data for each region. More specifically, we fit the time-variant parameters through three looping steps: first, we calculate the log-likelihood of each operator on the premise that the real reported confirmed cases obey a Poisson distribution with a mean of real reported confirmed cases on that day; second, we transform the log-likelihood to probability by exponentiation, to measure how much an operator is converging to the real epidemic history on that specific day; third, the operators will be weighted upon the corresponding probability, and a random sampling with put-back will be done to weed out operators deviating from real data.

Susceptible individuals could expand their social distancing to the extent that they would not be infected, at a social-distancing rate, and similarly, individuals who have social-distanced themselves could resume work at a work-resumption rate and become susceptible individuals again. Exposed infected individuals and symptomatic infected individuals could be isolated at a region-specific time-variant isolation rate. Once isolated, we assume that the individual would not infect others any more. We set the isolation period as seven days, as per the guidance from Public Health England, who suggest that people with COVID-19-like symptoms should self-isolate themselves for a 7-day medical observation since the time of symptom onset^19^. After isolation, if still do not recover, the isolated individuals will be hospitalized, tested, and reported. The asymptomatic cases could also be isolated through contact tracing. Considering Wuhan governmental interventions such as travel bans, social distancing, school and workplace closure, and contact tracing, mainly started on January 23rd, 2020^20^, thus, we assume no isolation or social distancing effects exist before January 23rd for both China and Western regions. A comparison of the moving trend of these parameters has been done between the Wuhan level, the Chinese average level, the United States level, and the Western average level.

### Data sources and processing

Previous studies reported that the Coronavirus firstly emerged in Wuhan, China in early December, 2019^21^. Thus, for Wuhan, Hubei Province excluding Wuhan, and other provinces in mainland China, data outlining total daily confirmed cases of COVID-19 were collected from December 10th, 2019 to May 1st, 2020, from the National Health Commission of the People’s Republic of China and the Health Commissions of distinct provinces^22^. For each selected Western country, data for the total number of daily confirmed cases were collected from the daily situation reports of the World Health Organization for the same period^23^. These data were fit in the extended stochastic meta-population model to estimate the dynamic trajectories of region-specific time-variant parameters, which would be the base for further scenario simulations.

Daily human mobility data between Wuhan, Hubei Province excluding Wuhan, and other provinces in mainland China were collected from the Baidu Migration Platform, which has now been closed due to stabilization of Chinese epidemic^24^. Mobility data from December 10th, 2019 to January 1st, 2020 were backwards inferred as 50% of the mobility on January 1st, 2020, which is moderately lower than the huge migration flows during the Chinese Spring Festival at the beginning of 2020.

Daily human mobility data between 13 Western countries (Switzerland, Sweden, Austria, France, the United Kingdom, Germany, Spain, Italy, Norway, the Netherlands, Belgium, Denmark and the United States) and between Western countries and Wuhan, Hubei Province excluding Wuhan, and other provinces in mainland China are inferred from seasonal flight data in 2019 from the European Union’s air passenger transport report of 12 European countries and origin and destination flight survey data from the Department of Transportation of the United States^25,26^.

Mobility data between Chinese regions and Western regions have been further corrected before application. First, based on daily flight statistics reports from Flight Manager^27^, from January 21st, 2020 to March 19th, 2020, to reduce the threat of epidemic spread, foreign airline companies substantially decreased daily numbers of international inbound and outbound flights in China by 87% on average compared with the average level before January 21st, 2020. Second, to control the growth of imported cases from abroad and avoid a second outbreak, the Civil Aviation Administration of China (CAAC) twice declared it would reduce the number of international flights, once on March 20th, 2020 and again on March 26th, 2020^28,29^. This is estimated to have decreased the number of international flights into and out of China by 37% and 85%, respectively. Moreover, since March 16th, 2020, to counter the spread of the novel coronavirus, the European Union passed a travel ban to restrict non-essential travel from non-EU countries into Europe^30^, so the human mobility data between the United States and European countries after March 16, 2020 are assumed to be 1% of the original.

### Trajectory predictions and scenario simulations

Trajectories of total reported cases, individuals infected but not hospitalized, new cases reported, total deaths, and new recoveries every day have been estimated for Wuhan, Hubei Province excluding Wuhan, and thirteen Western countries, from December 10th, 2020 to June 26th, 2020, not controlling for under-reporting. After controlling for under-reporting, two new trajectories are estimated: total reported cases controlling for under-reporting and individuals infected but not hospitalized controlling for under-reporting. The under-reporting rate of each country is based on a previous study which estimated under-reporting for China (24%) and numerous Western countries (Switzerland 22%, Sweden 6.3%, Austria 29%, France 5.1%, The United Kingdom 4.8%, Germany 25%, Spain 8.5%, Italy 7.3%, Norway 40%, The Netherlands 7.4%, Belgium 5.4%, Denmark 18%, and United States 13%)^11^.

In the simulation part, we have simulated three scenarios. First, to understand whether the rigorous strategy of Wuhan is more effective in curbing the spread of the epidemic, we examine a scenario where Western governments, from several time-points, simultaneously carry out intervention strategies in the same way as Wuhan, which is calculated as the average level of Wuhan in social-distancing and suspected-cases isolation during the first month after the shutdown in Wuhan. Second, to understand the significance of controlling imported cases from abroad in containing the epidemic, we have simulated a scenario where Western countries, from several time-points, simultaneously close their borders completely and ban all inbound or outbound mobility. Finally, we have simulated scenarios where less non-pharmaceutical interventions are in effect in China or Western countries to see how the epidemic would grow naturally without interventions.

Considering the COVID-19 epidemic may be already spreading globally since December, 2019^31^, the model was seeded with 2, 1, and 0.1 initial symptomatic infected individuals in Wuhan, Hubei Province excluding Wuhan, and in other Chinese provinces and Western countries on December 10th, 2019, with twice the number of exposed infected individuals.

### Analysis

Data were processed using Python version 3.7^32^. The model building and running, trajectories analysis, and visualization were done in R version 3.7.5^33^. A brief introduction of main model equations, model parameters, and parameter estimation methods is provided in the supplementary information for reference.

## Supplementary information

Supplementary Information.

## Data Availability

The data and code analyzed in this study are available on GitHub at https://github.com/1057499672/Globalized-stochastic-meta-population-SEIR-model.
