# Supplementary material for: SARS-Cov-2 trajectory predictions and scenario simulations from a global perspective: a modelling study
Source: Sci Rep. 2020 Oct 27;10:18319. doi: 10.1038/s41598-020-75332-x (PMC7591883; doi:10.1038/s41598-020-75332-x)
Supplement: Supplementary file 1 — Supplementary Information. [file 41598_2020_75332_MOESM1_ESM.pdf]

SARS-Cov-2 trajectory predictions and scenario simulations from a global perspective: a modelling study

Tianan Yang[1,2,3], Yexin Liu[1,2], Wenhao Deng[1,2], Weigang Zhao[1,2,3], Jianwei Deng[1,2,*]

[1] School of Management and Economics, Beijing Institute of Technology, Beijing, China

[2] Sustainable Development Research Institute for Economy and Society of Beijing, Beijing, China

[3] Center for Energy and Environmental Policy Research, Beijing Institute of Technology, Beijing, China

* Corresponding author (email: a1057499672@163.com)

Main model equations

Human mobility process

$$S_{i,\ t,outbound} = S_{i,t-1} * \frac{1}{Pop_i}\sum_{j=1}^{n} M_{i,j,t}$$

$$E_{1,i,\ t,outbound} = E_{1,i,t-1} * \frac{1}{Pop_i}\sum_{j=1}^{n} M_{i,j,t}$$

$$E_{2,,\ t,outbound} = E_{2,i,t-1} * \frac{1}{Pop_i}\sum_{j=1}^{n} M_{i,j,t}$$

$$S_{i,\ t,inbound} = \sum_{j=1}^{n} (M_{j,i,t} * \frac{1}{Pop_j}) * S_{j,t-1}$$

$$E_{1,i,\ t,inbound} = \sum_{j=1}^{n} (M_{j,i,t} * \frac{1}{Pop_j}) * E_{1,j,t-1}$$

$$E_{2,i,\ t,inbound} = \sum_{j=1}^{n} (M_{j,i,t} * \frac{1}{Pop_j}) * E_{2,j,t-1}$$

In the human mobility process, we let $M_{i,j,t}$ represent number of people travelling from place i to place j at day t. $S_{i,\ t,outbound}$ is the total number of susceptible people travelling outside from place i at day t. $E_{1,i,\ t,outbound}$ is the total number of exposed people in the first phase of incubation period travelled outside from place i at day t. $E_{2,i,\ t,outbound}$ is the total number of exposed people in the second phase of incubation period traveled outside from place i at day t. Similarly, $S_{i,\ t,inbound}$, $E_{1,i,\ t,outbound}$, and $E_{2,i,\ t,outbound}$ are respectively the total number of susceptible people, exposed people in the first phase of incubation period, and exposed people in the second phase of incubation period travelling into place i from other regions at day t.

Social distancing process

$$S_{i,\ t,to\ distancing} = S_{i,\ t-1} \ /\ Dis\_rate_{i,\ t}$$

$$Dis_{i,\ t,to\ S} = Dis_{i,\ t-1} \ /\ Sus\_rate_{i,\ t}$$

$$Dis_{i,\ t} = Dis_{i,\ t-1} + S_{i,\ t,to\ distancing} - Dis_{i,\ t,to\ S}$$

As the epidemic goes on, crisis awareness grows among people. Thus, some susceptible individuals at place i may choose social distancing on day t, whose speed is $Dis\_rate_{i,\ t}$, namely the average days needed for a susceptible individual to get social-distanced. Similarly, some people already in social distancing may choose to resume work and social activities, thus becoming susceptible individuals again, whose speed is $Sus\_rate_{i,\ t}$.

We assume social-distancing to be completely successful, protecting social-distanced individuals completely from any infection, and though it sounds radical in real life, it can prevent too sophisticated modelling and reach the same goal of intervention evaluations. We set the initial value of $Dis\_rate_{i,\ t}$ and $Sus\_rate_{i,\ t}$ to be seven days for all 45 regions, which during the modelling transmission process will change independently to fit real confirmation data of that region.

Isolation process

$E_{1,i,\ t,iso} = E_{1,i,\ t-1}\ /\ Iso\_rate_{i,\ t}$

$E_{2,i,\ t,iso} = E_{2,i,\ t-1}\ /\ Iso\_rate_{i,\ t}$

$I_{1,i,\ t,iso} = I_{1,,i,\ t-1}\ /\ Iso\_rate_{i,\ t}$

$I_{2,i,\ t,iso} = I_{2,i,\ t-1}\ /\ Iso\_rate_{i,\ t}$

$Iso_{i,\ t,to\,H} = Prob * \ Iso_{i,\ t-1}\ /\ Iso\_to\_H\_rate_{i,\ t}$

$Iso_{i,\ t} = \ Iso_{i,\ t-1} + \ E_{1,i,\ t,iso} + \ E_{2,i,\ t,iso} + \ I_{1,i,\ t,iso} + \ \ I_{2,i,\ t,iso}$ - $Iso_{i,\ t,to\,H}$

To curb the spread of virus, contract tracing and isolation of suspected and confirmed cases are necessary and have been broadly accepted. We model the isolation process using $Iso\_rate_{i,\ t}$*, and the former represents the average days needed for an exposed or symptomatic infective, as a suspected case, to be traced and isolated at place i and day t. The lower* $Iso\_rate_{i,\ t}$*, the more capable for a region to isolate suspected cases. We set the initial value of* $Iso\_rate_{i,\ t}$ *to be seven days for all 45 regions, which during the modelling transmission process will change independently and individually to fit real confirmation data of that region.*

$E_{1,i,\ t,iso}$ *means isolated infectives in the first phase of incubation period,* $E_{2,i,\ t,iso}$ *means isolated infectives in the second phase of incubation period,* $I_{1,i,\ t,iso}$ *means isolated infectives in the first phase of symptomatic period, and* $I_{2,i,\ t,iso}$ *means isolated infectives in the second phase of symptomatic period.*

In case of no sign of improvement for isolated people, they will be sent to hospital to get further treatment, whose rate is decided by $Iso_{i,\ t,to\,H}$*, referring to the average days needed for an unrecovered isolated infected individual to be sent to hospital. We set the value of* $Iso_{i,\ t,to\,H}$ *to be seven days as according to the latest guidance decreed by Public Health England*[1]*, which said that people who had coronavirus-related symptoms should isolate themselves at home for seven-days-watching before deciding whether to go to hospital. And Prob here is a weighted proportion of isolated people who do not recover during the isolation, which is determined as* $\Sigma w.\ e^{-(\frac{7}{average\ recovery\ period})}$*, where average recovery period here is different for people isolated in different phases and w is the weight for that group of people.*

Infection process

$$S_{i,t,to\,E\,1} = \frac{(S_{i,\ t-1} - \ S_{i,\ t,outbound} + \ S_{i,\ t,inbound} - \ S_{i,\ t,to\,distancing} + \ Dis_{i,\ t,to\,S})}{Pop_i}(\alpha E_{1,i,t-1} + E_{2,i,t-1} + \ I_{1,i,t-1} + I_{2,i,t-1} + \alpha E_{1,i,t,inbound} + E_{2,i,t,inbound} - \alpha E_{1,i,,t,outbound} - E_{2,i,t,outbound})\frac{R_0 * \varepsilon}{D_f}$$

$$E_{1,i,t,to\,E\,2} = \ (E_{1,i,\ t-1} - \ E_{1,i,\ t,outbound} + \ E_{1,i,\ t,inbound} \ - \ E_{1,i,\ t,iso})/(Inc_{rate_{i,\ t}}/2)$$

$$E_{2,i,t,to\,I\,1} = (1-\alpha)(E_{2,i,\ t-1} - \ E_{2,i,\ t,outbound} + \ E_{2,i,\ t,inbound} \ - \ E_{2,i,\ t,iso})/(Inc_{rate_{i,\ t}}/2)$$

$$I_{1,i,t,to\,I\,2} = \ (I_{1,i,\ t-1} \ - \ I_{1,i,\ t,iso})/(Inf_{rate_{i,\ t}}/2\ )$$

$$I_{2,i,t,to\,H} = \ (I_{2,i,\ t-1} \ - \ I_{2,i,\ t,iso})/(Inf_{rate_{i,\ t}}/2)$$

$S_{i,\ t} = \ S_{i,\ t-1} - \ S_{i,t,to\,E\,1}$

$E_{i,t,1} = \ E_{i,t-1,1} \ - \ E_{1,i,\ t,outbound} + \ E_{1,i,\ t,inbound} - E_{1,i,t,to\,E\,2} \ + S_{i,t,to\,E\,1} - E_{1,i,\ t,iso}$

$E_{i,t,2} = \ E_{i,t-1,2} \ - \ E_{2,i,\ t,outbound} + \ E_{2,i,\ t,inbound} - E_{2,i,t,to\,I\,1} + E_{1,i,t,to\,E\,2} - E_{2,i,\ t,iso}$

$I_{i,t,1} = \ I_{i,t-1,1} \ - I_{1,i,t,to\,I\,2} \ + \ E_{2,i,t,to\,I\,1} - I_{1,i,\ t,iso}$

---

1 https://www.gov.uk/government/publications/covid-19-stay-at-home-guidance/stay-at-home-guidance-for-households-with-possible-coronavirus-covid-19-infection

$$I_{i,t,2} = I_{i,t-1,2} - I_{2,i,t,to\,H} + I_{1,i,t,to\,I\,2} - I_{2,i,\,t,\,iso}$$

$S_{i,t,to\,E\,1}$ is the number of susceptible people infected at place i and day t, which is calculated as the product term of proportion of susceptible people in the whole population, the number of infectives able to infect other people, and $\frac{R_0*\varepsilon}{D_f}$, the average people that an infected individual can infect in one day in a wholly susceptible population. As we have taken human mobility into account, in calculating the proportion of susceptible people in the whole population, the outbound susceptible people should be excluded while the inbound susceptible people from elsewhere should be included, and in the same ideology, the susceptible people getting social-distanced yesterday should be excluded while social-distanced people getting susceptible yesterday by resuming work should be included. Likewise, the number of exposed infected individuals in the incubation period, $E_{i,t,1}$ and $E_{i,t,2}$, should exclude those moving out and include those moving in. α is the proportion of asymptomatic cases, which we set as 7.5%[2].

Here we actually put a simplified version of the original conditional equations concerning the symptomatic infected individuals, including $I_{i,t,1}$ and $I_{i,t,2}$, as we have assumed that infected individuals could move freely across regions only before Wuhan shutdown.

$\frac{R_0*\varepsilon}{D_f}$ represents the average cases generated by a typical infectious person daily in a full susceptible population, where R_0 refers to the basic reproduction number, which we set at 2.5[3], while ε is the random walk number generated from gamma distribution with standard deviation set at 0.3395.[4] D_f refers to the whole infective period which in our model equals 7.24 days, equating to half of the incubation period $Inc\_rate_{i,\,t}$ multiplying asymptomatic proportion, plus half of the incubation period, plus the symptom onset period $Inf\_rate_{i,\,t}$, set at 3.8 days[5].

Exposed infectives in the first phase may turn into second phase, and from the second phase may turn into first-phase symptomatic infectives, both at a rate of $Inc_{rate_{i,\,t}}/2$. Symptomatic infectives may turn into second phase from the first phase, which will finally turn to hospitalization, both at a rate of $Inf_{rate_{i,\,t}}/2$.

Recovery process

$$Hospital_{i,\,t} = Hospital_{i,\,t-1} + I_{2,i,t,to\,H} * \left(1 - Die\_prop_{i,t}\right) + Iso_{i,\,t,to\,H} * \left(1 - Die\_prop_{i,t}\right) - H_{i,\,t,to\,recovery1}$$

$$H_{i,\,t,to\,recovery1} = Hospital_{i,\,t-1} \,/\, \left(Rec\_rate_{i,\,t}/2\right)$$

---

2 Koo, J. R. et al. Interventions to mitigate early spread of SARS-CoV-2 in Singapore: a modelling study. Lancet Infect Dis. 20, 678-688 (2020).

3 Kucharski AJ1, Russell TW2, Diamond C2, Liu Y2, Edmunds J2, Funk S2, Eggo RM2, et al. Early dynamics of transmission and control of COVID-19: a mathematical modelling study. Lancet Infect Dis. 2020 Mar 11. pii: S1473-3099(20)30144-4. doi: 10.1016/S1473-3099(20)30144-4.

4 Kucharski AJ1, Russell TW2, Diamond C2, Liu Y2, Edmunds J2, Funk S2, Eggo RM2, et al. Early dynamics of transmission and control of COVID-19: a mathematical modelling study. Lancet Infect Dis. 2020 Mar 11. pii: S1473-3099(20)30144-4. doi: 10.1016/S1473-3099(20)30144-4.

5 Zhang, J. et al. Evolving epidemiology and transmission dynamics of coronavirus disease 2019 outside Hubei province, China: a descriptive and modelling study. *Lancet Infect Dis*. 20, 793-802 (2020). .

$$Rec_{1,i,\ t,to\ recovery2} = Rec_{1,i,\ t-1} /\ (Rec\_rate_{i,\ t}/2)$$

$$Rec_{1,i,t} = Rec_{1,i,t-1} + H_{i,\ t,to\ recovery1} - Rec_{1,i,\ t,to\ recovery2}$$

$$Rec_{2,i,t} = Rec_{2,i,t-1} + Rec_{1,i,\ t,to\ recovery2}$$

In the recovery process, we assume that patients would experience two phases until final recoveries, and $Rec\_rate_{i,\ t}$ *is the average time from hospitalization to recovery, set at 18.2 days.* $Die\_prop_{i,t}$ *is the mortality rate for COVID-19, which we set as the average mortality rate for each region upon May 1st, 2020.*

Death process

$$To\_death_{i,t} = To\_death_{i,t-1} + I_{2,i,t,to\ H} * Die\_prop_{i,t} + Iso_{i,\ t,to\ H} * Die\_prop_{i,t} - New\_Death_{i,\ t}$$
$$New\_Death_{i,\ t} = To\_death_{i,\ t-1} /\ (Die\_rate_{i,\ t}/2)$$
$$Death_{i,t} = Death_{i,t-1} + New\_Death_{i,\ t}$$

In the death process, we also assume that patients would experience two phases until final deaths, and $Die\_rate_{i,\ t}$ *is the average time from hospitalization to death, set at 14.7 days*[6]*.* $Die\_prop_{i,t}$ *is the mortality rate for COVID-19, which we set as the average mortality rate for each region upon May 1st, 2020.*

Reporting process

$$To\_report_{i,\ t} = W_{i,\ t-1} * Rep\_rate_{i,\ t}$$
$$W_{i,\ t} = W_{i,\ t-1} + I_{2,i,t,to\ H} + Iso_{i,\ t,to\ H} - To\_report_{i,\ t}$$
$$Report_{i,\ t} = Report_{i,\ t-1} + To\_report_{i,\ t}$$

We assume that infected people will be tested and reported after hospitalization. However, this process consumes time. $W_{i,\ t-1}$ *is the number of cases waiting to be reported at day t-1,* and $Rep\_rate_{i,\ t}$ *refers to the reporting rate, the inverse of reporting time. We set reporting time, the time needed for an infected individual to be official reported after hospitalization, as 4.5 days before Jan 27, 2020, and 2.8 days after Jan 27, 2020*[7]*.*

Main parameters

Model parameters	*Constant or variable*	*Initial value*	*References*
R0 (reproduction number)	*variable*	*2.5*	Kucharski, A. J. et al. Early dynamics of transmission and control of COVID-19: a mathematical modelling study. *Lancet Infect Dis*. **20**, 553-558 (2020).
social-distancing rate	*variable*	*7 days*	
work-resuming rate	*variable*	*7 days*	

[6] Zhou, F. et al. Clinical course and risk factors for mortality of adult inpatients with COVID-19 in Wuhan, China: a retrospective cohort study. *Lancet.* 395, 1054- 1062 (2020).

[7] Zhang, J. et al. Evolving epidemiology and transmission dynamics of coronavirus disease 2019 outside Hubei province, China: a descriptive and modelling study. Lancet Infect Dis. 20, 793-802 (2020).

isolation rate	*variable*	*7 days*	
incubation period	*constant*	*6.4 days*	Prem,K. et al. The effect of control strategies to reduce social mixing on outcomes of the COVID-19 epidemic in Wuhan, China: a modelling study. *Lancet Public Health*. **5**, e261-e270 (2020).
symptom-onset period	*constant*	*3.8 days*	Zhang, J. et al. Evolving epidemiology and transmission dynamics of coronavirus disease 2019 outside Hubei province, China: a descriptive and modelling study. *Lancet Infect Dis*. **20**, 793-802 (2020).
dying period (non-survivors)	*constant*	*14·7 days*	Zhou, F. et al. Clinical course and risk factors for mortality of adult inpatients with COVID-19 in Wuhan, China: a retrospective cohort study. *Lancet.* **395**, 1054- 1062 (2020).
recovery period (survivors)	*constant*	*18.2 days*	Zhou, F. et al. Clinical course and risk factors for mortality of adult inpatients with COVID-19 in Wuhan, China: a retrospective cohort study. *Lancet.* **395**, 1054- 1062 (2020).
proportion of asymptomatic cases	*constant*	*7.50%*	Meng, H. et al. CT imaging and clinical course of asymptomatic cases with COVID-19 pneumonia at admission in Wuhan, China. *J Infect*. **81**, e33-e39 (2020).
official reporting delay	*constant*	*4.5 days before Wuhan shutdown, 2.8 days after Wuhan shutdown*	Zhang, J. et al. Evolving epidemiology and transmission dynamics of coronavirus disease 2019 outside Hubei province, China: a descriptive and modelling study. *Lancet Infect Dis*. **20**, 793-802 (2020).
under-reporting rate	*constant*	*stated in the manuscript*	Russel, T. W. et al. Using a delay-adjusted case fatality ratio to estimate under-reporting. *Center for Mathematical Modelling of Infectious Diseases.*

			https://cmmid.github.io/topics/covid19/global_cfr_estimates.html (2020).

Parameter estimation methods

We divide our parameter estimation methods into two sections: fitting part and predicting part.

1. *Fitting part*

In the fitting algorithm, we combine the stochastic process, log-likelihood estimation, and evolutionary algorithm to estimate the dynamic change of R0, the dynamic change of isolation rate, work-resuming rate, and social-distancing rate.

The stochastic process consists of thousands of independent operators, and each operator is an independent system, including a whole set of parameters, like regional-specific time-variant R0, social-distancing rate, isolation rate, and work-resuming rate. Each operator will be initialized on the first day of modelling: for instance, R0 will be initialized as 2.5, and work-resuming rate, isolation rate, and social-distancing rate will be initialized at 7 days for an individual to resume work, to be isolated if infected, to get social-distanced.

Then, the parameters and epidemic trajectories of each operator will fluctuate randomly and individually. For instance, the initial R0 will fluctuate obeying a normal distribution with a mean of 0 and a standard variance of 0.3395; and the initial isolation rate, work-resuming rate, and social-distancing rate will fluctuate obeying a normal distribution with a mean of 0 and a standard variance of 0.2.

In this way, due to different parameters on the next day, operators will develop into different epidemic trajectories. However, we just want the trajectories running close to the real trajectories, which are the real history epidemic data published online from each nation or region.

To eliminate operators deviating from the real epidemic history, and maintain those converging into the real epidemic history, we have further adopted log-likelihood estimation method and evolutionary algorithm. More specifically, it includes three steps:

First, we use log-likelihood estimation method to estimate the log-likelihood of each operator on the premise that the reported confirmed cases obey a Poisson distribution with a mean of real reported confirmed cases on that day, then we transform the log-likelihood to probability by exponentiation, to measure how much an operator is converging to the real epidemic history on that specific day, then we allocate weights for different operators according to their degree of converging to real epidemic history (the probability), where the more an operator deviates from the real history, the less weight it will be allocated.

Second, we will sample new operators randomly with put-back (pub-back means an operator can be sampled more than once) from the original operators according to their weights. In this way, an evolution process will be accomplished, and the operators selected will be those converging to the real epidemic history.

Third, we will replace the original operators with the new operators, as well as their parameters. In this way, a fitting process for one day will be realized.

When the model is running to the final day (for instance, May 1st), a group of operators will be sampled randomly according to their weights from the last day back to the first day. In this way, we realize the fitting process and gain the regional-specific time-variant trajectories of R0, social-distancing rate, work-resuming rate, and isolation rate

2. *Predicting part*

Unlike a combination of stochastic process and evolutionary algorithm in the fitting part, in the predicting part, we employ a combination a stochastic process and dynamic planning. Dynamic planning here means that we use the first-day data to predict the second-day data, and use the second-data to predict the third-day data, where each day of the whole trajectory is decided by the day before. However, the only difference between this part and the fitting part is that we use different weighting methods. In the fitting part, weights will be allocated according to the degree of converging to real epidemic history, while in the predicting part, the weights will be allocated according to the degree of being close to the medium of all operators on that day. In this way, we can avoid a complete random-walking prediction while keeping regional-specific parameters fluctuating differently by time with proper randomness.

Model flowchart

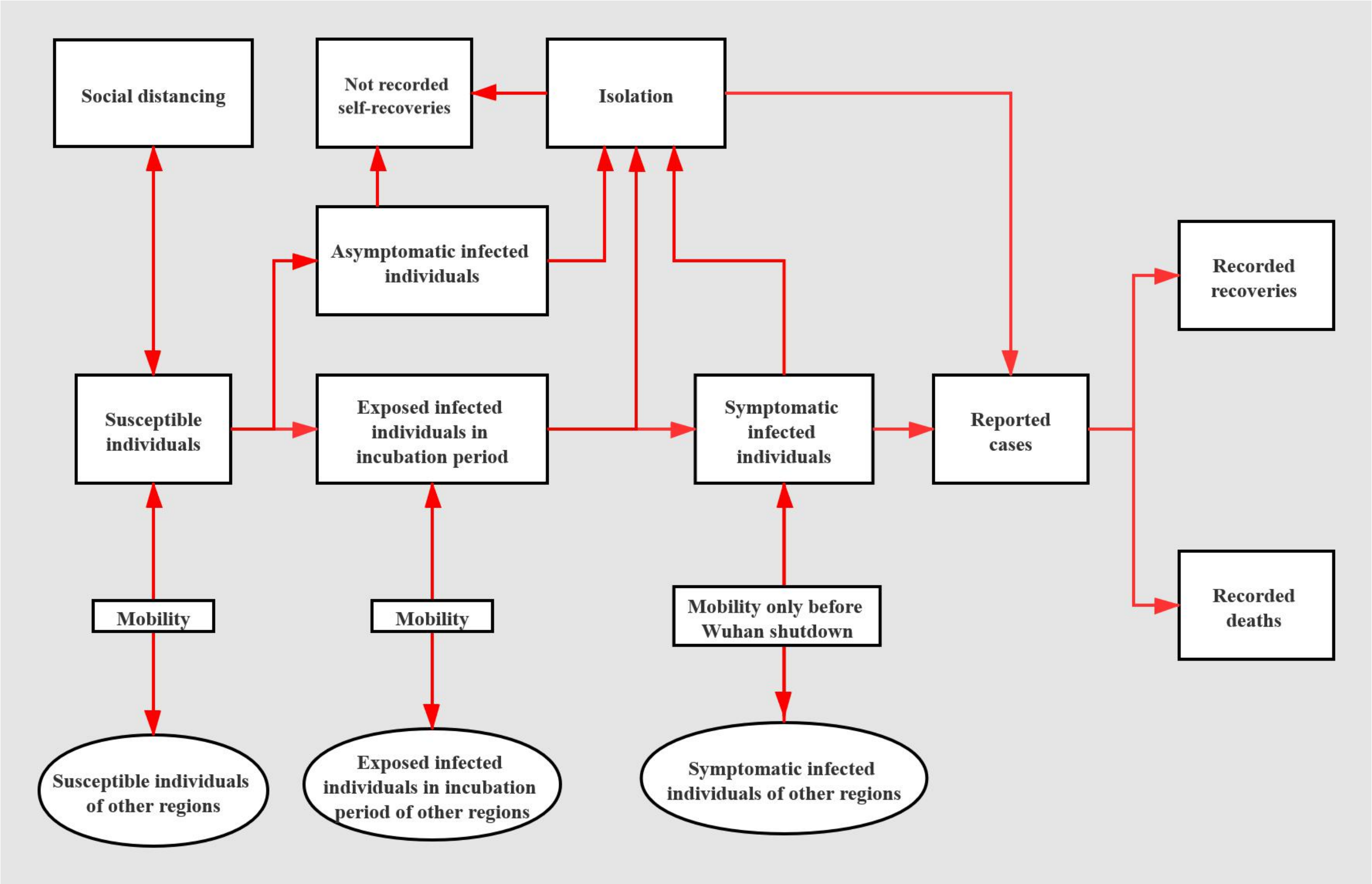

After careful scrutiny, we have revised the model flowchart a little to make it accurately and clearly conform to our model.

The model flowchart shows an extended SEIR model. We stratify the natural infective process into four stages: susceptible individuals, exposed infected individuals in the incubation period, symptomatic infected individuals, and removed individuals (who either recovered or died).

However, different from tradition SEIR model, we included 7.5% asymptomatic cases, and integrated human mobility process as well as non-pharmaceutical interventions into model: for the human mobility, susceptible individuals and exposed infected cases in incubation period could move freely across regions, and symptomatic infected cases could move across regions only before Wuhan shutdown; for the social-distancing interventions, we assume that susceptible individuals could expand their social distancing to the extent that they would not be infected, at a social-distancing rate, and similarly, individuals who have

social-distanced themselves could resume work at a work-resumption rate and become susceptible individuals again; for the isolation interventions, asymptomatic cases, exposed infected individuals in the incubation period, as well as symptomatic infected individuals could be isolated at a region-specific time-variant isolation rate. After isolation, if still do not recover during 7 days' watch, the isolated individuals will be hospitalized, tested, and reported; for the intercity-travel ban, we can flexibly adjust the human mobility matrix.

After an incubation period of 6.4 days, exposed infected individuals, if not isolated, will develop into symptomatic infected individuals. And symptomatic infected individuals, if not isolated, would turn into hospitalization, get tested and reported after a symptom-onset period of 3.8 days. Once being hospitalized, the only end is either recovery or death, we assume both of which will remove the case.
